# Diagnosis and treatment of pulmonary *Aspergillus* infection secondary to empyema complicated with lung cancer: a case report

**DOI:** 10.3389/fmed.2025.1660190

**Published:** 2025-08-13

**Authors:** Chen Zhu, Xiuli Liu, Ying Gu, Yuyao Shen, Tingshu Jiang

**Affiliations:** Department of Respiratory and Critical Care Medicine, Yantai Yuhuangding Hospital, Yantai, China

**Keywords:** lung cancer, empyema, BALF, bronchoscopic, Invasive Pulmonary *Aspergillosis*

## Abstract

**Background:**

This study aimed to explore the clinical characteristics, diagnosis, and management of Invasive Pulmonary *Aspergillosis* (IPA) complicating empyema in a patient with lung cancer.

**Case presentation:**

We analyzed the clinical manifestations, bronchoscopic findings, and therapeutic approaches, including anti-infective and anti-tumor treatments, in a patient diagnosed with IPA-associated empyema and lung cancer. Histopathological examination of bronchoscopic biopsy specimens confirmed pulmonary adenocarcinoma, while bronchoalveolar lavage fluid cultures identified *Aspergillus fumigatus*. Following the development of secondary empyema, comprehensive anti-infective and anti-tumor therapies were administered, leading to disease control. IPA-associated empyema complicating lung cancer represents a rare clinical scenario.

**Conclusion:**

In patients presenting with IPA, the potential coexistence of lung cancer should be considered. An integrated approach combining antifungal therapy and active anti-tumor treatment is essential for managing such cases.

## Background

*Aspergillus* spp. cause disease through respiratory inhalation of spores (1). Fungal pneumonia can present with similar symptoms and radiological findings to lung cancer (2), often associated with chemotherapy-induced immunosuppression and high-dose glucocorticoid use (1). Even with adequate antifungal treatment, Invasive Pulmonary *Aspergillosis* (IPA) is associated with poor prognosis in different populations, including critically ill patients with Corona Virus Disease 2019 (COVID-19) (3) or influenza (4, 5). Some estimates have also included IPA complicating lung cancer, estimated from one large study in China at 2.6% (6). It represent a huge disease burden. The concurrent management of empyema secondary to *Aspergillus* infection, incorporating antibacterial, antifungal, and antitumor therapies, remains underreported. This case report details a patient with empyema complicating lung cancer secondary to IPA, analyzing clinical features, treatment strategies, and outcomes.

## Case presentation

The patient was admitted to the hospital on May 27, 2023, with a chief complaint of “fever and cough for 7 days.” Seven days prior to admission, the patient developed fever without an apparent cause, reaching a peak temperature of 38.7°C, accompanied by paroxysmal coughing and expectoration of white sputum. He received intravenous treatment with cefuroxime and azithromycin at a local hospital for 3 days; however, his symptoms did not significantly improve, prompting referral to our department for further evaluation and management. The outpatient evaluation established a preliminary diagnosis of pneumonia, prompting hospitalization. No significant past medical history but reported a 20-pack-year smoking history (10 cigarettes/day). The patient has no family history of genetic disorders or mental illness.

Physical Examination: Temperature 36.5°C, Pulse 123 beats/min, Respiratory Rate 20 breaths/min, Blood Pressure 136/97 mmHg.

**Figure 1:**
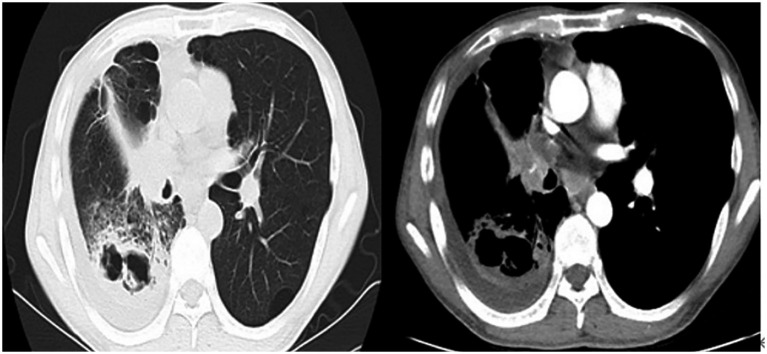
Chest computed tomography (CT) scan dated May 27, 2023.

Auxiliary tests included: Complete blood count + C-reactive protein (CRP) + Serum amyloid A (SAA): White blood cell count 23.12 × 10^9/L, hypersensitive C-reactive protein 88.99 mg/L, serum amyloid A 178.07 mg/L; Procalcitonin 0.127 ng/L, Aspergillus-specific antibody > 500 AU/mL. The patient’s Partial Pressure of Oxygen in Arterial Blood was 68 mmHg. A chest computed tomography (CT) scan demonstrated cavitary changes in the right lower lobe of the lung, right pleural effusion, and mediastinal lymphadenopathy (Figure 1).

He was diagnosed with: Pneumonia with pulmonary cavity formation, mediastinal lymphadenopathy, and pleural effusion.

**Figure 2:**
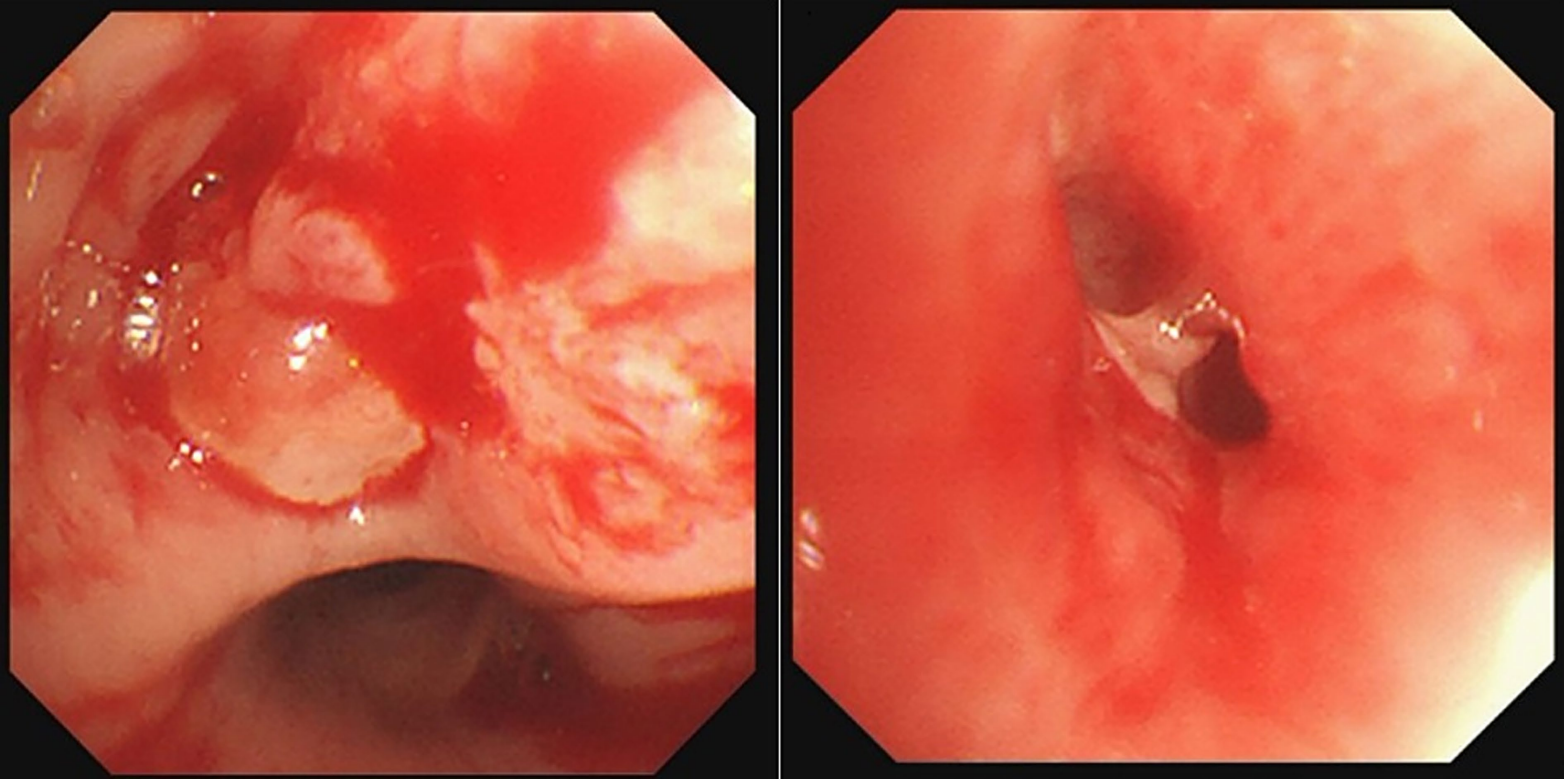
Bronchoscopy revealed an exophytic lesion in the right middle lobe bronchus (RB4) completely obstructed the lumen, Purulent secretion and lumen stenosis were observed in the right lower lobe.

**Figure 3:**
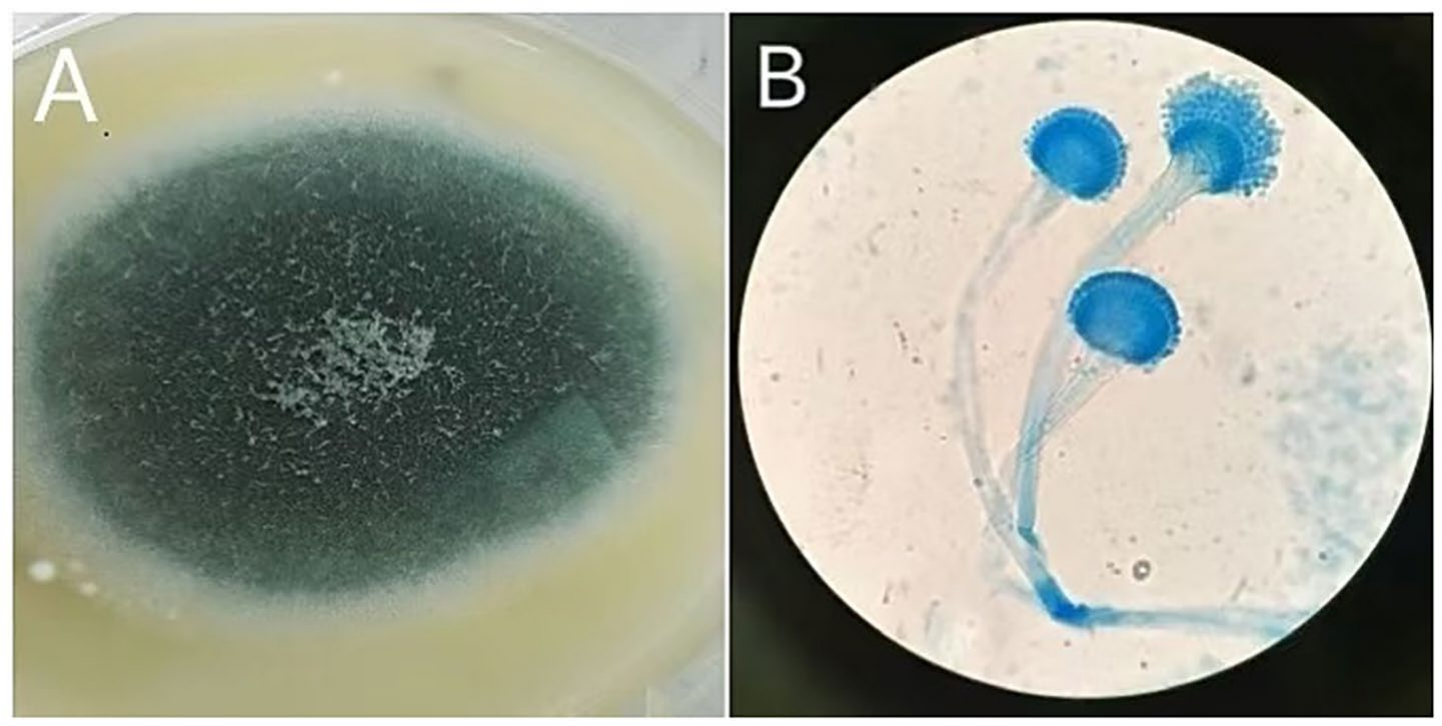
Macroscopic and microscopic features of *Aspergillus*. **(A)** On Sabouraud Dextrose Agar, the colony appears white and velvety, with a gray-green surface and a colorless or yellow-brown reverse. **(B)** Microscopic examination reveals smooth conidiophores with a flask-shaped apex, and conidia are densely arranged at approximately two-thirds of the conidiophore’s length, consistent with *Aspergillus* infection.

**Figure 4:**
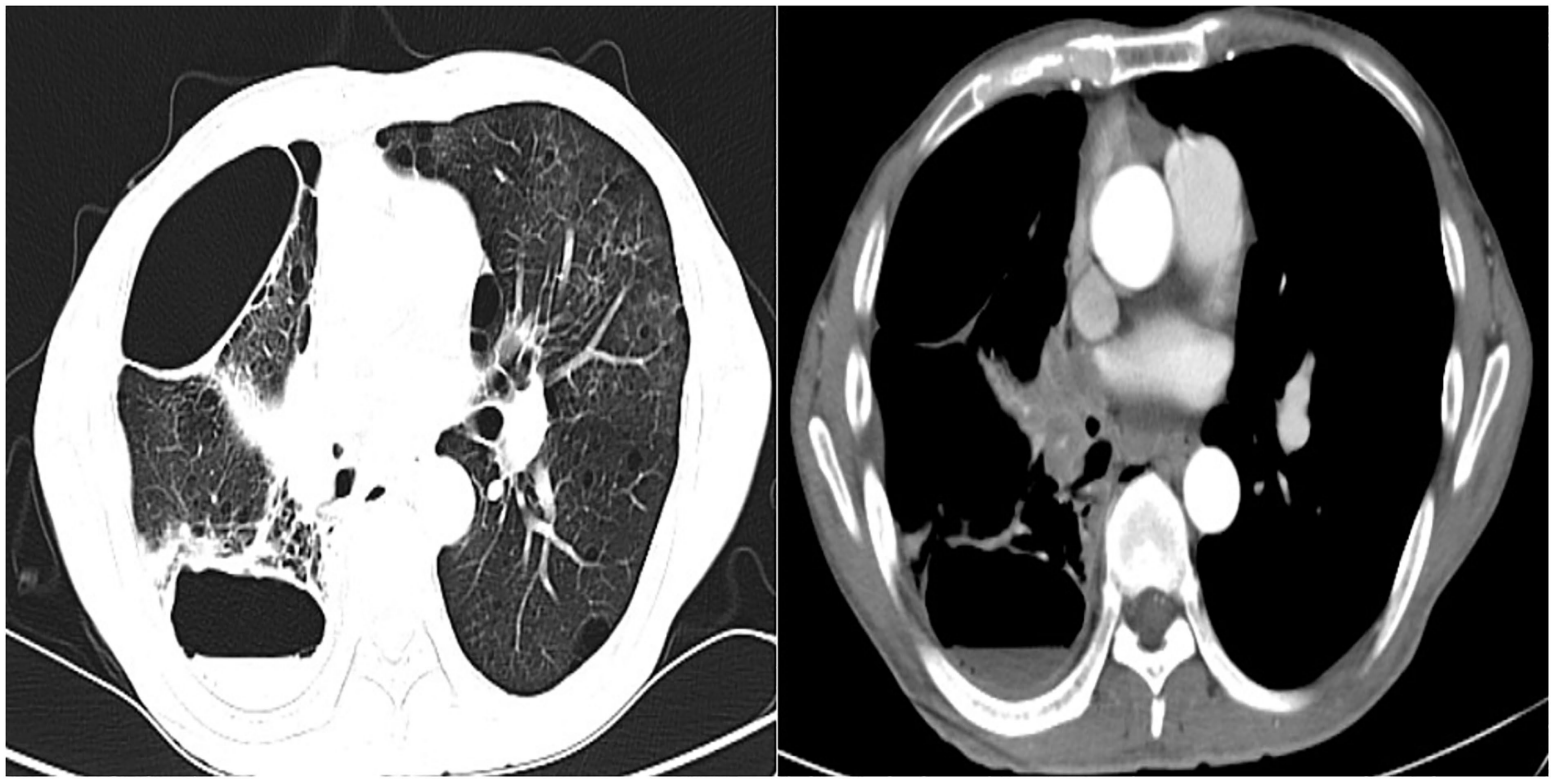
Follow-up chest CT scan review (June 21, 2023).

Procedure of diagnosis and treatment: After hospitalization, the patient received ultrasound-guided pleural catheter drainage and biopsy, The pleural fluid met Light’s criteria for exudate (protein 42.36 g/L, LDH 682 U/L, Glu 5.4 mmol/L, ADA 9.5 U/L, CEA987ng/ml), histopathology showed dense collagen deposition with fibroblast proliferation, consistent with reactive fibrosis. No tumor cells were found in the pleural fluid. The patient underwent fiberoptic bronchoscopy on May 30, 2023 (Figure 2). An exophytic lesion was identified in the right middle lobe bronchus (RB4), completely obstructing the lumen, demonstrating marked vascularity with active bleeding during forceps biopsy attempts. Purulent secretion obstruction was found in the right lower lobe, lumen stenosis was noted. Bronchoalveolar Lavage was performed in the right lower lobe. Bronchoscopy pathology obtained from a lung biopsy revealed an epithelial-derived malignancy. Immunohistochemistry results were consistent with a diagnosis of lung adenocarcinoma. Next-generation sequencing (NGS) identified a pathogenic Neurofibromin 1, for which the targeted therapeutic drug, selumetinib, was not accepted by the patient due to its high cost. BAL fluid was sent for fungal culture, *Aspergillus fumigatus* was cultured on Sabouraud Dextrose Agar (Figure 3). The isolation and identification of Aspergillus fumigatus from BAL samples was performed by culturing the samples on Sabouraud Dextrose Agar (SDA) at 28°C for 5 to 7 days. Pure colonies were obtained using dilution plating and streaking methods. The macroscopic appearance of *A. fumigatus* colonies was white and velvety, with a gray-green surface and a colorless or yellow-brown reverse, The microscopic examination revealed typical conidiophores and conidia chains. Molecular identification was carried out by extracting genomic DNA, amplifying the ITS or *β*-tubulin gene regions via PCR, and sequencing the products. The sequences were then compared to GenBank databases to confirm the identification (Accession Number: MH101019). Administer voriconazole 200 mg every 12 h for antifungal treatment. Voriconazole was discontinued due to drug-induced hallucinations and substituted with itraconazole (200 mg/day), amphotericin B (5 mg bid nebulization), and piperacillin-tazobactam sodium (4.5 g q8h) for anti-infective treatment. Despite these measures, the patient experienced recurrent fever, prompting an upgrade to meropenem 1 g q8h from June 5 onwards, and discontinuation of itraconazole oral therapy. Micafungin 100 mg/day antifungal treatment and methylprednisolone 40 mg qd anti-inflammatory treatment were initiated from June 9 to June 18, stabilizing the patient’s body temperature. Cefoperazone-Sulbactam (2, 1) sodium 3 g q8h anti-infection treatment was administered from June 13 to June 20. The patient underwent a thorough baseline tumor staging evaluation and was diagnosed with lung adenocarcinoma, staged as AJCC8th cT4N3M1a, stage IV. The first cycle of chemotherapy was administered starting on June 23, consisting of pemetrexed 0.8 g on day 1 and cisplatin 30 mg on day 1. Post-chemotherapy, the patient developed fever again. A follow-up chest CT on June 21 (Figure 4) showed persistent abnormalities. Ultrasound-guided right pleural puncture catheter drainage was performed from July 22, extracting purulent fluid and pleural fluid with the following characteristics: specific gravity >1.018, Rivalta test (+), mononuclear cells 7.6%, multiple nuclear cells 92.4%, nucleated cell count 204,242 × 10^6/L; carcinoembryonic antigen (CEA): carcinoembryonic antigen 37.4 ng/mL; Pleural fluid biochemistry: total protein 17.27 g/L, glucose <0.60 mmol/L, lactate dehydrogenase 29,005 U/L, adenosine deaminase 143.7 U/L, The pleural effusion has evolved into a purulent empyema, suggesting secondary infection (Table 1). The culture of the pleural effusion was negative for microbial growth. Antibiotics were adjusted to moxifloxacin 0.4 g combined with meropenem 1 g q8h for anti-infection treatment, along with Micafungin 100 mg/day for antifungal treatment. The patient’s temperature stabilized gradually, and the thoracic drainage tube was subsequently removed. Post-discharge, the patient continued itraconazole capsule 200 mg/day. The antifungal agents were withdrawn following completion of the standardized 6-month therapeutic course. The patient received 6 cycles of chemotherapy with pemetrexed 0.8 g d1 + cisplatin 30 mg d1 q21d. Chemotherapy was discontinued after 6 cycles due to compliance issues. After 6 months of oral itraconazole antifungal treatment, the patient discontinued the medication and has been regularly followed up in the respiratory outpatient clinic. No recurrence was observed during the 12-month follow-up, indicating disease stability. Follow-up chest CT was reviewed on September 10, 2024 (Supplementary Figure S1). The clinical treatment course for both *Aspergillus* infection and lung cancer is shown in Supplementary Figure S2.

**Table 1:** Comparative analysis of pleural fluid between May 18, 2023, and July 22, 2023.

Pleural fluid analysis	May 18, 2023	July 22, 2023
Color	Red	Tawny
Transparency	Slightly turbid	Slightly turbid
Clot	None	None
Red Blood Cells	+++	+++
Neutrophils (%)	46.5%	7.6%
Lymphocytes (%)	53.5%	92.4%
Nucleated cell count	1,402 × 10^6^/L	204,242 × 10^6^/L
Rivalta’s test	+	+
ADA (U/L)	9.5	143.7
Total protein (g/L)	42.36	17.27
LDH (U/L)	682	29,050
CEA (ng/ml)	987	0.94
Glucose (mmol/L)	5.40	<0.60

## Discussion and conclusions

IPA predominantly affects immunocompromised hosts, though our case demonstrates its occurrence in a lung cancer patient prior to immunosuppressive therapy. To our knowledge, few biology-based and treatment-stratified studies have explored IPA’s prevalence in patients with advanced-stage lung cancer and their survival or IPA risk factors (7). Pulmonary *Aspergillosis* is uncommon in patients with intact immune function but may occur in those with compromised immunity due to conditions such as lung cancer. Studies have reported an incidence of 18.7% for co-occurring lung cancer and fungal infections (8). In lung cancer patients, local mucosal immune barriers are often compromised, leading to tissue necrosis, which facilitates *Aspergillus* colonization and subsequent local invasion and infection (4). Lung cancer represents a high-risk factor for *Aspergillus* infection, particularly in patients undergoing radiotherapy and chemotherapy, as these treatments can weaken the immune system, predisposing patients to opportunistic infections. Clinicians should be alert to underlying malignant disease if airway *Aspergillus* infection is suspicious in a patient without strong risk factors for invasive fungal disease. On the other hand, when lung cancer is coexisting with airway necrotizing *Aspergillosis*, clinicians should properly manage these two diseases simultaneously (2). However, cases have been observed where *Aspergillus* infection occurred prior to any treatment. The mortality rate for *Aspergillus*-related empyema in lung cancer patients remains high (45–60%), and combined antifungal and antitumor therapy is crucial for improving outcomes (9). Early bronchoscopy and pleural fluid analysis are critical for diagnosis. Combined antifungal therapy (e.g., voriconazole and amphotericin B) with chemotherapy showed improved survival rates (10). Patients who received combined therapy had significantly better survival rates compared to those treated with antifungal therapy alone (11). In this case, the patient presented with fever and cough, and imaging revealed cavity lesions in the lower lobe of the right lung. Fiberoptic bronchoscopy identified new organisms in the middle lobe of the right lung, and a biopsy confirmed the pathological diagnosis. Despite aggressive antibacterial and antifungal therapy, the patient developed recurrent fever and elevated white blood cell counts. This suggested a leukemoid reaction. Hormonal anti-inflammatory therapy was administered, after which the patient’s temperature stabilized and their condition improved. Wang Qin noted that factors such as smoking, emphysema, bronchial diseases, diabetes, chronic obstructive pulmonary disease (COPD), and prolonged chemotherapy cycles increase the risk of pulmonary fungal infections in advanced non-small cell lung cancer patients (12). Therefore, it is crucial to manage co-existing conditions, enhance overall resistance, and select less toxic chemotherapy regimens to minimize the risk of pulmonary fungal infections. In this case, the chest CT imaging findings suggest that the patient has comorbid COPD, a known risk factor for *Aspergillus* infection. The patient developed secondary empyema due to IPA. Alongside antibacterial and antifungal treatments, a reduced platinum-based chemotherapy regimen was administered. After adequate drainage and anti-infection measures, the patient’s temperature was controlled, and her condition stabilized. Following effective infection control, 6 cycles of regular chemotherapy were initiated. Fungal empyema is rare, accounting for less than 1% of all empyema cases. While Candida is the most common causative agent, *Aspergillus* is rarely implicated (13, 14). The mortality rate associated with *Aspergillus* empyema can be as high as 45% (9). A study conducted in Japan demonstrated that the cause of death in lung cancer patients with concomitant Chronic Pulmonary *Aspergillosis* who received anticancer drug treatments and effective antifungal treatment was lung cancer progression. Further large-scale studies are needed to identify the effect of Chronic Pulmonary *Aspergillosis* in patients with lung cancer (15). In this case, the patient received reduced-intensity chemotherapy alongside antifungal therapy. Post-chemotherapy, empyema secondary to IPA was diagnosed. Timely thoracic puncture and drainage, along with appropriate antibiotics, controlled the infection. Imaging showed cavity lesions in the lower lobe of the right lung with crescent signs, suggestive of an *Aspergillus* cavity. Although no *Aspergillus* was detected in pleural fluid cultures, likely due to low positive rates, the imaging evolution and treatment response supported the diagnosis of empyema secondary to *Aspergillus* infection. *Aspergillus* may be more likely to colonize and infect lung tissue rather than fluid-filled spaces like the pleural cavity, where it might not proliferate as effectively. The pleural fluid in cases of empyema is often exudative and may have high protein content, which can complicate the culture process by interfering with fungal growth or making it harder to isolate the fungus. Li et al. (16) reported a mortality rate of 51.1–60% in lung cancer patients with *Aspergillus* infections, highlighting the high mortality and suboptimal therapeutic outcomes. Case report is limited by its single-patient design, limiting the generalizability of outcomes. Future studies with larger cohorts are required to validate these observations. Fiberoptic bronchoscopy plays a critical role in diagnosing suspected lung cancer while ruling out fungal infections. Clinical practice should focus on comprehensive management, addressing both malignancy and complicating infections, with individualized treatment plans tailored to each patient’s unique clinical profile.

## Data Availability

The datasets presented in this study can be found in online repositories. The names of the repository/repositories and accession number(s) can be found in the article/Supplementary material.

## Supplementary material

SUPPLEMENTARY FIGURE S1 Reevaluation of Chest CT Scan Conducted on September 10, 2024.

SUPPLEMENTARY FIGURE S2 The clinical treatment timeline for both *Aspergillus* infection and lung cancer.
