# Correction: Diagnosis and treatment of pulmonary *Aspergillus* infection secondary to empyema complicated with lung cancer: a case report

**DOI:** 10.3389/fmed.2025.1685872

**Published:** 2025-08-29

**Authors:** Chen Zhu, Xiuli Liu, Ying Gu, Yuyao Shen, Tingshu Jiang

**Affiliations:** Department of Respiratory and Critical Care Medicine, Yantai Yuhuangding Hospital, Yantai, China

**Keywords:** lung cancer, empyema, BALF, bronchoscopic, Invasive Pulmonary *Aspergillosis*

The reference details for (2), (9), (10), and (11) were erroneously written as:

2. Wu T, Li P, Wang M, Wang Q, et al. Pulmonary solid tumor with coexisting pulmonary aspergillosis: case reports and literature review. Clin Respir J. 2017;11(1):3-12. doi: 10.1111/crj.12345
9. Smith J Brown A Johnson R Kirkevold Ø Halvorsen TO. Fungal empyema in lung cancer patients: a retrospective analysis. J Thorac Oncol. (2023) 18:123–30. doi: 10.1016/j.jtho.2023.01.012
10. Lee H Kim S Park J Park JE Jung SS Na JO et al. Invasive pulmonary aspergillosis complicated by empyema in non-small cell lung cancer: a case series. Mycopathologia. (2022) 187:259–69. doi: 10.1007/s11046-022-00623-y, PMID: 35314920
11. Wang X Liu Y Chen Z Zhou C Zhang J Chen Q et al. Combined antifungal and antitumor therapy for *aspergillus* empyema in lung cancer: a retrospective analysis. Front Oncol. (2022) 12:876543. doi: 10.3389/fonc.2022.876543

It should be Wu et al. (2), Nigo et al. (9), Koulenti et al. (10), and Marr et al. (11).

The correct references are as follows:

2. Wu T, Li P, Wang M, Wang Q, Shi Y, Su X. Pulmonary solid tumor with coexisting pulmonary aspergillosis: case reports and literature review. *Clin Respir J*. (2017) 11:3–12. doi: 10.1111/crj.12294
9. Nigo M, Vial MR, Munita JM, Jiang Y, Tarrand J, Jimenez CA, et al. Fungal empyema thoracis in cancer patients. *J Infect*. (2016) 72:615–21. doi: 10.1016/j.jinf.2016.02.014
10. Koulenti D, Garnacho-Montero J, Blot S. Approach to invasive pulmonary aspergillosis in critically ill patients. *Curr Opin Infect Dis*. (2014) 27:174–83. doi: 10.1097/QCO.0000000000000043
11. Marr KA, Boeckh M, Carter RA, Kim HW, Corey L. Combination antifungal therapy for invasive aspergillosis. *Clin Infect Dis*. (2004) 39:797–802. doi: 10.1086/423380

The original version of this article has been updated.
