# Supplementary figures and images for: Diagnosis and treatment of pulmonary Aspergillus infection secondary to empyema complicated with lung cancer: a case report
Source: Front Med (Lausanne). 2025 Aug 13;12:1660190. doi: 10.3389/fmed.2025.1660190 (PMC12380570; doi:10.3389/fmed.2025.1660190)

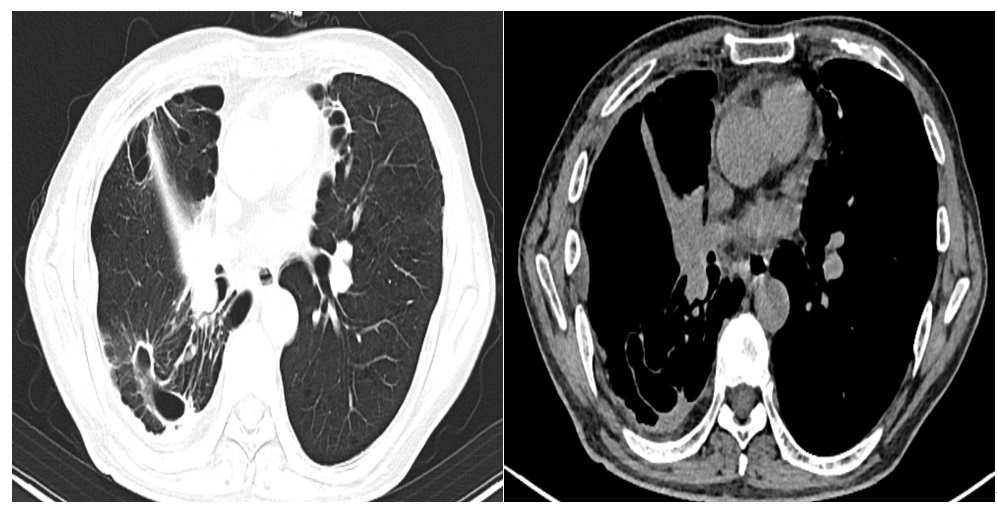

Supplement: SUPPLEMENTARY FIGURE S1 — Reevaluation of Chest CT Scan Conducted on September 10, 2024. [file Image_1.JPEG]

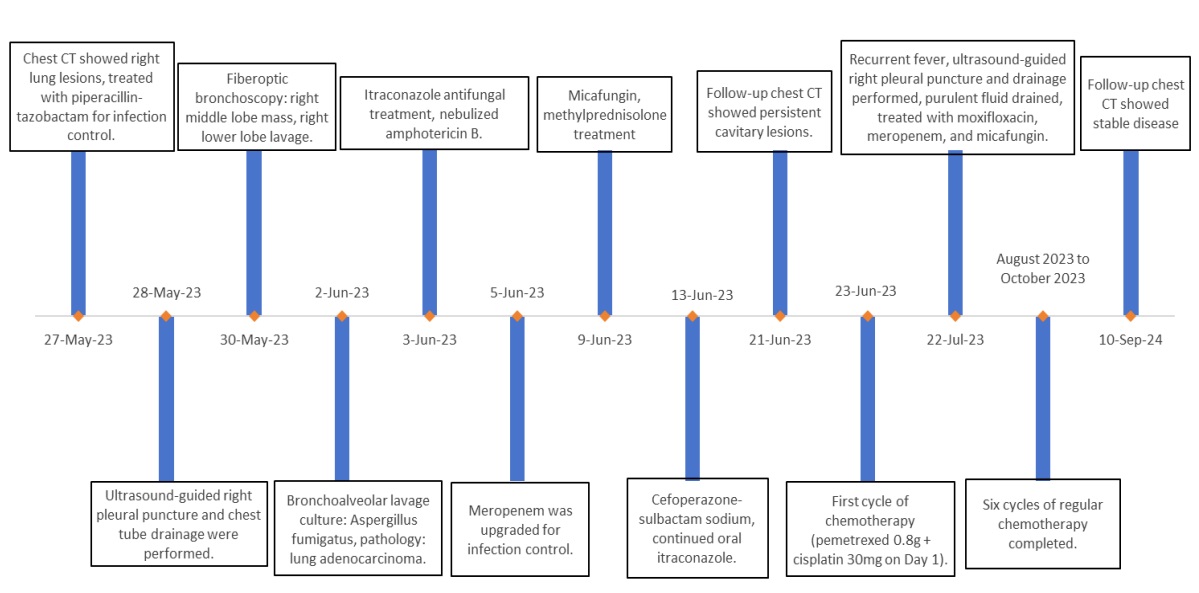

Supplement: SUPPLEMENTARY FIGURE S2 — The clinical treatment timeline for both Aspergillus infection and lung cancer. [file Image_2.JPEG]
